# Correction: El-Hefnawy et al. Fabrication of Nanofibers Based on Hydroxypropyl Starch/Polyurethane Loaded with the Biosynthesized Silver Nanoparticles for the Treatment of Pathogenic Microbes in Wounds. *Polymers* 2022, *14*, 318

**DOI:** 10.3390/polym16213099

**Published:** 2024-11-04

**Authors:** Mohamed E. El-Hefnawy, Sultan Alhayyani, Mohsen M. El-Sherbiny, Mohamed I. Sakran, Mohamed H. El-Newehy

**Affiliations:** 1 Department of Chemistry, Rabigh College of Sciences and Arts, King Abdulaziz University, Jeddah 21589, Saudi Arabia; salhayyani@kau.edu.sa; 2 Marine Biology Department, Faculty of Marine Sciences, King Abdulaziz University, Jeddah 21589, Saudi Arabia; ooomar@kau.edu.sa; 3 Biochemistry Department, Faculty of Science, University of Tabuk, Tabuk 47731, Saudi Arabia; msakran@ut.edu.sa; 4 Department of Chemistry, Faculty of Science, Tanta University, Tanta 31527, Egypt; 5 Department of Chemistry, College of Science, King Saud University, Riyadh 11451, Saudi Arabia

There was an error in the original publication [1]. The authors regret that incorrect images had been placed in Figure 2, Figure 3 and Figure 8 due to a data processing error.

The corrected versions of the images are displayed below.

**Figure 2:**
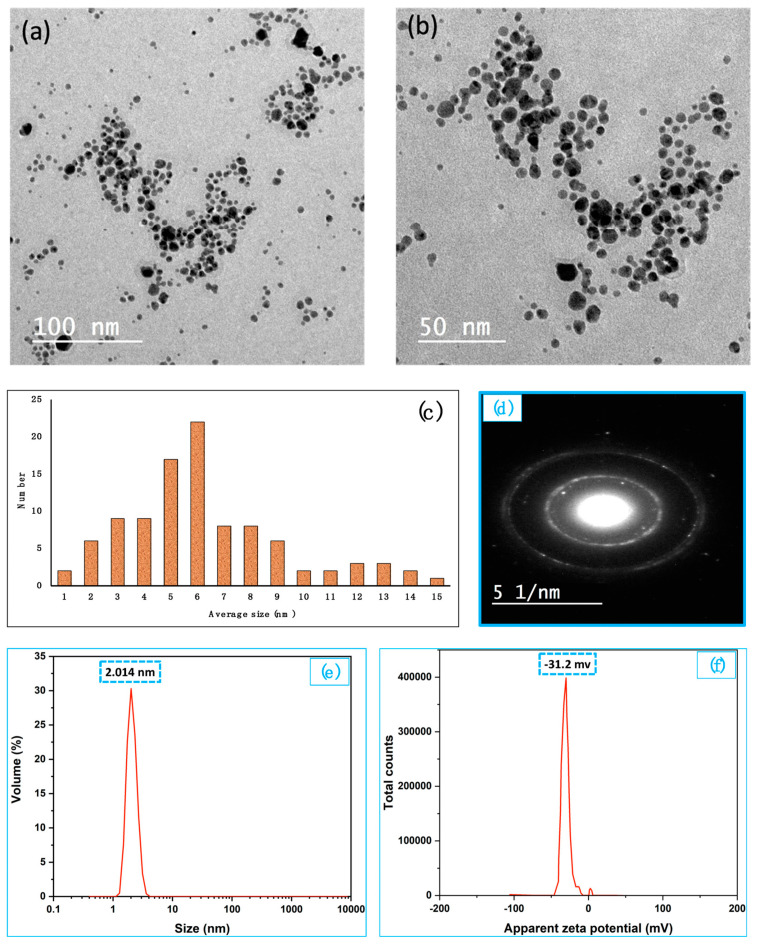
(**a**,**b**) TEM of AgNPs. (**c**) distribution size calculated from TEM©ages (**d**) SAED, (**e**) hydrodynamic particle size and (**f**) zeta potential of AgNPs.

**Figure 3:**
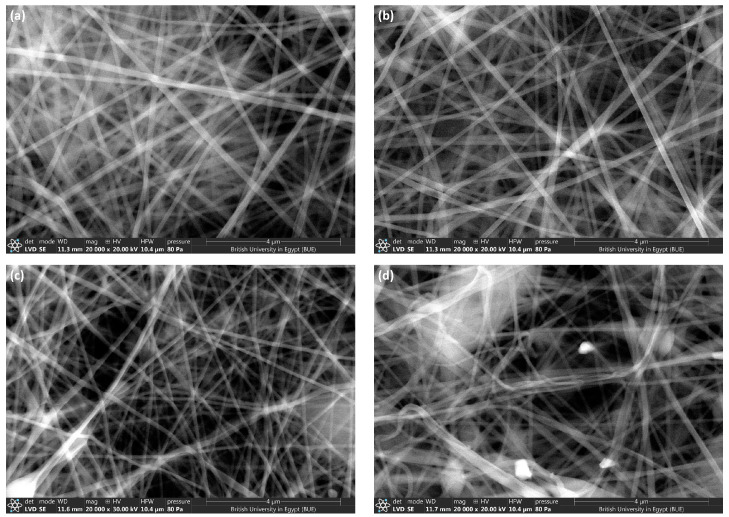
SEM images of (**a**) AgNPs-0@NFs, (**b**) AgNPs-1@NFs, (**c**) AgNPs-2@NFs and (**d**) AgNPs-3@NFs.

The SEM images were taken at high magnification (20,000×) using QUARTO S THERMOFISHER (USA). All SEM images (Figure 3a–d) of HPS/PU nanofibers unloaded and loaded with different concentrations of AgNPs were uniformly prepared with no significant beads, uniform and smooth nanofibers (Figure 3b–d). The formation of bead-free nanofibers occurred due to the high spinnability of PU and the low concentration of the utilized HPS (1). It is remarkable that the addition of AgNPs has no impact on the morphology of nanofibers in terms of short fibers or bead fibers. Moreover, it can be assumed that most of the small spherical AgNPs were incorporated into the porous structure of the prepared nanofibers.

**Figure 8:**
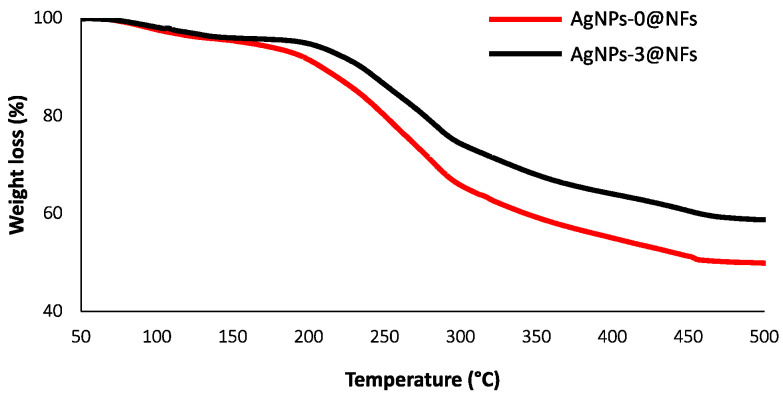
TGA of nanofibrous sheets with and without AgNPs: AgNPs-0@NFs and AgNPs-3@NFs.

Thermal gravimetric analysis (TGA) was used to outline the thermal stability of the nanofiber sheet. AgNPs-0@NFs and AgNPs-3@NFs were selected for TGA analysisthis. The weighted samples were analyzed from room temperature to 500 °C. From Figure 8, it is clearly seen that the nanofibers loaded with AgNPs had higher thermal stability than the nanofibers without AgNPs, signifying that AgNPs served as a filler and improved the polymer’s excellent thermalstability (1).

The authors apologize for any inconvenience caused and state that the scientific conclusions are unaffected. This correction was approved by the Academic Editor. The original publication has also been updated.
