# Supplementary material for: Fabrication of Nanofibers Based on Hydroxypropyl Starch/Polyurethane Loaded with the Biosynthesized Silver Nanoparticles for the Treatment of Pathogenic Microbes in Wounds
Source: Polymers (Basel). 2022 Jan 13;14(2):318. doi: 10.3390/polym14020318 (PMC8779972; doi:10.3390/polym14020318)
Supplement: Supplementary file 1 [file polymers-14-00318-s001.zip › polymers-1543372-supplementary-done.pdf]

polymers

Supplementary data

Fabrication of nanofibers based on hydroxypropyl starch/polyurethane loaded with the biosynthesized silver nanoparticles for the treatment of pathogenic microbes in wounds

Mohamed E. El-Hefnawy[1*], Sultan Alhayyani[1], Mohsen M. El-Sherbiny[2], Mohamed I. Sakran[3,5] and Mohamed H. El-Newehy[4,5*]

[1] Department of Chemistry, Rabigh College of Sciences and Arts, King Abdulaziz University, Jeddah 21589, Saudi Arabia

[2] Marine Biology Department, Faculty of Marine Sciences, King Abdulaziz University, Jeddah 21589, Saudi Arabia

[3] Biochemistry Department, Faculty of Science, University of Tabuk, Tabuk, Saudi Arabia

[4] Department of Chemistry, College of Science, King Saud University, Riyadh 11451, Saudi Arabia.

[5] Department of Chemistry, Faculty of Science, Tanta University, Tanta 31527, Egypt.

* Corresponding author: E-mail: malhefnawy@kau.edu.sa (M.E.E.) and melnewehy@ksu.edu.sa (M.H.E.)

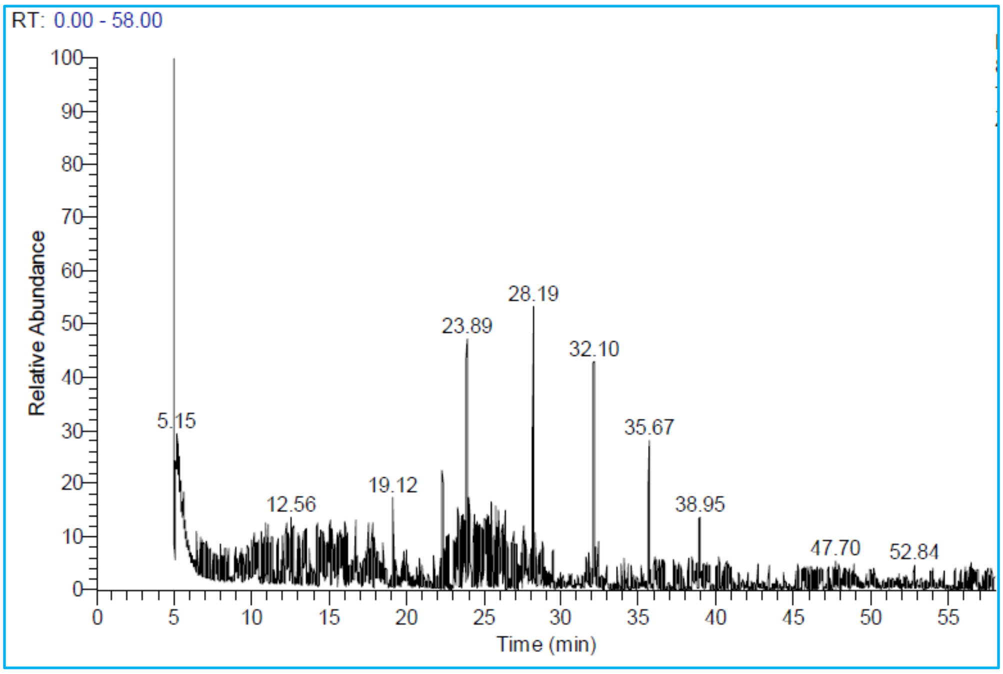

Figure S1. GC-MS chromatogram of *Nerium oleander* extract.

Table S1. Chemical compositions of *Nerium oleander* extract.

No.	R_t	Area%[a]	M.W.	M.F.	Identified compounds	Class/Category
1	5.15	12.04	266	$C_{18}H_{34}O$	5-Octadecenal	Fatty aldehydes
2	19.12	2.15	224	$C_{16}H_{32}$	1-Hexadecene	Unbranched alkene
3	22.34	2.71	206	$C_{13}H_{18}O_2$	2-Allyl-5-t-butylhydroquinone	Hydroquinone derivatives
4	23.33	1.21	692	$C_{44}H_{44}N_4O_4$	N,N'-Dicyclohexyl-1,7-dipyrrolidinylperylene-3,4: 9,10-tetracarboxylicacidbisimide	Pyrrolidine derivatives
5	23.89	5.70	490	$C_{35}H_{70}$	17-Pentatriacontene	Long-chain alkane
6	24.04	1.70	242	$C_{13}H_{22}O_4$	Oxalic acid, allyl octyl ester	Diester
7	25.77	1.38	760	$C_{48}H_{56}O_8$	Methoxychromene precocene tetramer	Chromenes
8	25.90	1.51	678	$C_{45}H_{58}O_5$	7,13,19,25-Tetratertbutyl-27,28,29,30-tetrahydroxy-2,3-bishomo-3-oxacalix[4]arene	Tetratertbutyl derivatives
9	27.45	1.39	688	$C_{42}H_{56}O_8$	2,3-Diacetoxy-6,7,10,11-tetrapentyloxytriphenylene	Triphenylenes
10	28.19	5.92	200	$C_{13}H_{28}O$	1-Tridecanol	Long-chain fatty alcohol
11	28.31	1.21	618	$C_{44}H_{90}$	Tetratetracontane	Long-chain alkane
12	32.10	5.0	266	$C_{19}H_{38}$	(cis)-2-nonadecene	Unsaturated aliphatic hydrocarbons
13	35.67	3.15	382	$C_{26}H_{54}O$	1-Hexacosanol	Long-chain fatty alcohol
14	38.95	2.05	326	$C_{22}H_{46}O$	1-Docosanol	Long-chain fatty alcohol
		**T% 47.12**				

Rt: Retention time; **M.W.:** Molecular weight; **M.F.:** Molecular formula.
